# Resonant X-ray photo-oxidation of light-harvesting iron (II/III) *N*-heterocyclic carbene complexes

**DOI:** 10.1038/s41598-021-01509-7

**Published:** 2021-11-12

**Authors:** Robert H. Temperton, Meiyuan Guo, Giulio D’Acunto, Niclas Johansson, Nils W. Rosemann, Om Prakash, Kenneth Wärnmark, Joachim Schnadt, Jens Uhlig, Petter Persson

**Affiliations:** 1 MAX IV Laboratory, Lund University, Box 118, 221 00 Lund, Sweden; 2 School of Physics and Astronomy, University of Nottingham, Nottingham, NG7 2RD UK; 3 Lund Institute of Advanced Neutron and X-ray Science, IDEON Building: Delta 5, Scheelevägen 19, 223 70 Lund, Sweden; 4 Division of Chemical Physics, Department of Chemistry, Lund University, Box 124, 221 00 Lund, Sweden; 5 Division of Synchrotron Radiation Research, Department of Physics, Lund University, Box 118, 221 00 Lund, Sweden; 6 Department of Chemistry, Centre for Analysis and Synthesis, Lund University, Box 124, 221 00 Lund, Sweden; 7 Division of Theoretical Chemistry, Department of Chemistry, Lund University, Box 124, 221 00 Lund, Sweden

**Keywords:** Organometallic chemistry, Chemical physics, Light harvesting

## Abstract

Two photoactive iron *N*-heterocyclic carbene complexes ${[\hbox {Fe}^{{{\rm{II}}}}(\hbox {btz})_2(\hbox {bpy})]^{2+}}$ and ${[\hbox {Fe}^{{\rm{III}}}(\hbox {btz})_3]^{3+}}$, where btz is 3,3’-dimethyl-1,1’-bis(p-tolyl)-4,4’-bis(1,2,3-triazol-5-ylidene) and bpy is 2,2’-bipyridine, have been investigated by Resonant Photoelectron Spectroscopy (RPES). Tuning the incident X-ray photon energy to match core-valence excitations provides a site specific probe of the electronic structure properties and ligand-field interactions, as well as information about the resonantly photo-oxidised final states. Comparing measurements of the Fe centre and the surrounding ligands demonstrate strong mixing of the Fe ${\hbox {t}_{{\rm{2g}}}}$ levels with occupied ligand $\pi$ orbitals but weak mixing with the corresponding unoccupied ligand orbitals. This highlights the importance of $\pi$-accepting and -donating considerations in ligand design strategies for photofunctional iron carbene complexes. Spin-propensity is also observed as a final-state effect in the RPES measurements of the open-shell $\hbox {Fe}^{{\rm{III}}}$ complex. Vibronic coupling is evident in both complexes, where the energy dispersion hints at a vibrationally hot final state. The results demonstrate the significant impact of the iron oxidation state on the frontier electronic structure and highlights the differences between the emerging class of $\hbox {Fe}^{{\rm{III}}}$ photosensitizers from those of more traditional $\hbox {Fe}^{{\rm{II}}}$ complexes.

## Introduction

Iron-based transition metal complexes are attractive for the development of earth-abundant solar energy conversion technologies^1–3^, but traditional $\hbox {Fe}^{{\rm{II}}}$ polypyridyl complexes suffer from rapid losses of the excited state energy from initially excited metal-to-ligand charge transfer (MLCT) states to low-energy metal-centred (MC) states^4^. Recent progress with strong sigma-donating ligands have improved the photophysical properties of iron complexes dramatically^5–13^. Surprisingly, $\hbox {Fe}^{{\rm{II}}}$ and $\hbox {Fe}^{{\rm{III}}}$ *N*-heterocyclic carbenes (NHC) have demonstrated long-lived charge-transfer (CT) states^14,15^ capable of driving interfacial electron injection^16^ and bimolecular photo-redox reactions in solution^17^, respectively. Additionally, a photo-active bimetallic $\hbox {Fe}^{{\rm{II}}}$/${\hbox {Co}^{{\rm{III}}}}$ compound has been recently reported^18^. The photofunctionality of the $\hbox {Fe}^{{\rm{III}}}$ complexes is particularly noteworthy given that the photo-excited Ligand-to-Metal CT (LMCT) excited states have only rarely shown useful photochemical properties for $\hbox {d}^5$ transition metal systems^8^.

**Figure 1:**
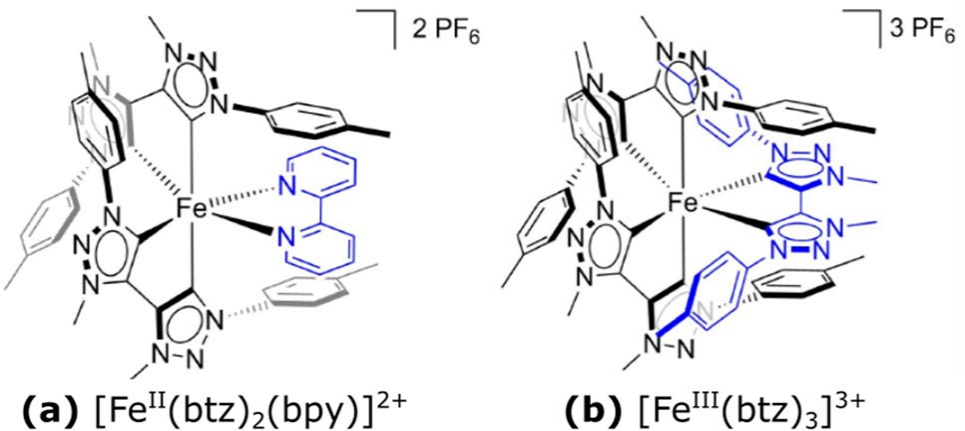
Schemes of the two Fe-NHC complexes studied: **(a)** ${[\hbox {Fe}^{{{\rm{II}}}}(\hbox {btz})_2(\hbox {bpy})]^{2+}}$ and **(b)** ${[\hbox {Fe}^{{\rm{III}}}(\hbox {btz})_3]^{3+}}$, where btz = 3,3’-dimethyl-1,1’-bis(p-tolyl)-4,4’-bis(1,2,3-triazol-5-ylidene) and bpy = 2,2’-bipyridine.

**Figure 2:**
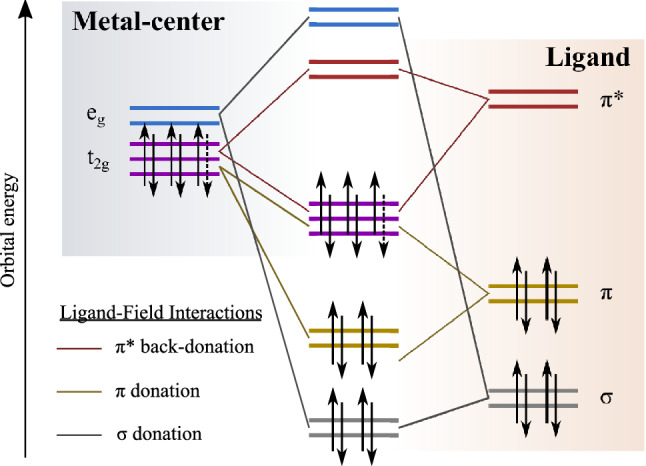
Schematic representation of the metal-ligand interactions in the quasi-octahedral ligand field-splitting convention for $\hbox {Fe}^{{\rm{II}}}$ and $\hbox {Fe}^{{\rm{III}}}$ complexes with closed shell $3\hbox {d}^6$ and open shell $3\hbox {d}^5$ electronic configurations respectively. The dashed arrow indicates the electron in the ${\hbox {t}_{{\rm{2g}}}}$ that is only present in the $\hbox {d}^6$ case. The left and right of the figure shows the molecular orbitals originating from the metal ion and ligand respectively. The combination of these orbitals in an octahedral geometry is depicted in the centre of the figure.

Here we investigate the $\hbox {Fe}^{{\rm{II}}}$ and $\hbox {Fe}^{{\rm{III}}}$ carbene complexes ${[\hbox {Fe}^{{{\rm{II}}}}(\hbox {btz})_2(\hbox {bpy})]^{2+}}$ and ${[\hbox {Fe}^{{\rm{III}}}(\hbox {btz})_3]^{3+}}$, shown in Fig. 1, where btz is 3,3’-dimethyl-1,1’-bis(p-tolyl)-4,4’-bis(1,2,3-triazol-5-ylidene and bpy is 2,2’-bipyridine^14,19^. The electronic structure properties of such low-spin octahedral complexes are generally understood from ligand field theory^20^. A strong, symmetric ligand field on the central Fe atom causes the 3*d* orbitals to split in energy into occupied ${\hbox {t}_{{\rm{2g}}}}$ and unoccupied ${\hbox {e}_{{\rm{g}}}}$ metal-centred levels as shown in Fig. 2. Key metal-ligand bonding interactions typically include destabilisation of the metal ${\hbox {e}_{{\rm{g}}}}$ levels by sigma donation from the ligands, while the metal ${\hbox {t}_{{\rm{2g}}}}$ levels are destabilised by $\pi$ donation and stabilised by $\pi ^*$ back-donation^7,21^.

The electronic structure properties of transition metal complexes can be studied using X-ray spectroscopy methods including X-ray absorption spectroscopy (XAS)^22,23^ and photoelectron spectroscopy (PES)^24–26^. XAS and emerging time-resolved X-ray techniques^27^ have contributed to a better understanding of the properties of iron–carbene photosensitisers in terms of electronic structure^28–31^, excited-state deactivation pathways^32^, and excited-state dynamics such as hot branching^33^ and wave packet oscillations^34^. We have also used resonant photoelectron spectroscopy (RPES) to probe site-selective orbital interactions in ${[\hbox {Fe}^{{\rm{III}}}(\hbox {btz})_3]^{3+}}$^35^.

**Figure 3:**
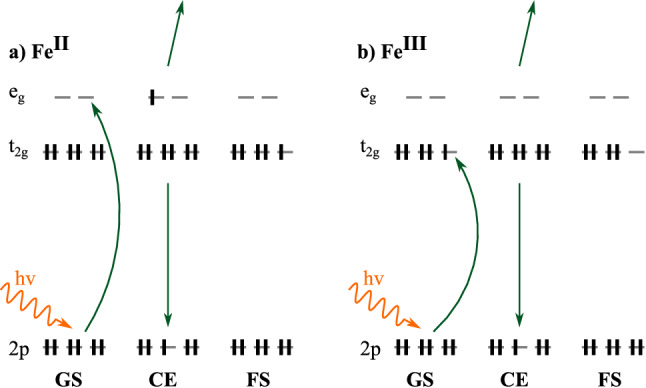
Scheme of the participant decay process following the lowest iron-centred Fe 2*p* resonant excitation for **(a)** $\hbox {Fe}^{{\rm{II}}}$ and **(b)** and $\hbox {Fe}^{{\rm{III}}}$ octahedral complexes. The initial/ground state (GS), the core-excited (CE) intermediate state, and the oxidised final state (FS) after participant decay are shown, where green arrows indicate the excitation and decay processes.

In RPES, the incident photon energy is tuned to match a resonant transition allowing the resulting “participant” decay channel to be measured^36^. This is outlined in Fig. 3 for the lowest energy metal-centred Fe 2*p* resonant excitation for low-spin $\hbox {Fe}^{{\rm{II}}}$ and $\hbox {Fe}^{{\rm{III}}}$ complexes. Participant decay describes an autoionisation process in which one electron is emitted and the other one fills the core hole. The emitted electron manifests as an enhancement of the valence photoemission signal corresponding to the participating occupied molecular orbital. RPES therefore connects the initial, intermediate (core-to-valence excited) and final (oxidised) states of a system, as described by the Kramers–Heisenberg equation^37^. The other notable decay channel present in RPES data is “resonant Auger”/“spectator” decay, in which the core-excited electron does not actively participate in the Auger decay step that fills the core hole^38^. It is however important to highlight that whilst the “particpant”/“spectator” nomenclature is useful when discussing spectral features in RPES, it has limitations and in some cases the distinction is not uniquely defined^39^.

Several metal complexes have been studied with RPES, including the Ru centred light-harvesting complexes “N3”^40,41^ and “N719”^42^. RPES has also been used to probe the metal centre of the ferricyanide and ferrocyanide model complexes^43^. However, RPES studies have rarely probed both the metal and ligand contributions allowing electronic structure features from each to be placed on a common binding energy axis. Recent studies of hexacyano cobaltate^44^ and ${[\hbox {Fe}^{{\rm{III}}}(\hbox {btz})_3]^{3+}}$^35^ suggest this is a useful experimental strategy providing information about the overlap of metal/ligand derived orbitals as well the mixing of occupied and unoccupied states. Here, we expand this strategy to include a comprehensive RPES study of an open-shell transition metal complex.

This work uses RPES to compare the complexes ${[\hbox {Fe}^{{{\rm{II}}}}(\hbox {btz})_2(\hbox {bpy})]^{2+}}$ and ${[\hbox {Fe}^{{\rm{III}}}(\hbox {btz})_3]^{3+}}$ in terms of the Fe oxidation state influence on the ligand field and electronic structure properties. For ${[\hbox {Fe}^{{{\rm{II}}}}(\hbox {btz})_2(\hbox {bpy})]^{2+}}$, Fe 2*p* RPES is used to probe the central iron atom whilst N 1*s* and C 1*s* RPES probes the btz and bpy ligands. For ${[\hbox {Fe}^{{\rm{III}}}(\hbox {btz})_3]^{3+}}$, we use Fe 2*p* RPES to probe the metal centre, which we connect to our recently published RPES study of the ligand interactions^35^. The similar ligand environments in the two complexes allow a good comparison of $\hbox {Fe}^{{\rm{II}}}$ and $\hbox {Fe}^{{\rm{III}}}$ oxidation states. Additionally, it is noteworthy that the valence-ionised final state in RPES distinguishes this technique from the related technique of Resonant Inelastic X-ray Scattering (RIXS), which has successfully been used to investigate transition metal complexes^41,45^, including the highly relevant ferrous and ferric hexacyanide^46^. The RIXS process is the photon-in/photon-out analogue of RPES (photon-in/electron-out), and as such the final state is not oxidised. The resonant photo-oxidation accomplished in RPES therefore provides unique opportunities to understand the electronic properties of consecutive higher oxidation states in a single experiment. This is of particular relevance to transition metal complexes, whose redox properties are critical to their application in devices, whilst higher oxidation state variants of a complex may not be stable enough for many conventional characterisation methods.

## Results

### ${[\hbox {Fe}^{{{\rm{II}}}}(\hbox {btz})_2(\hbox {bpy})]^{2+}}$

**Figure 4:**
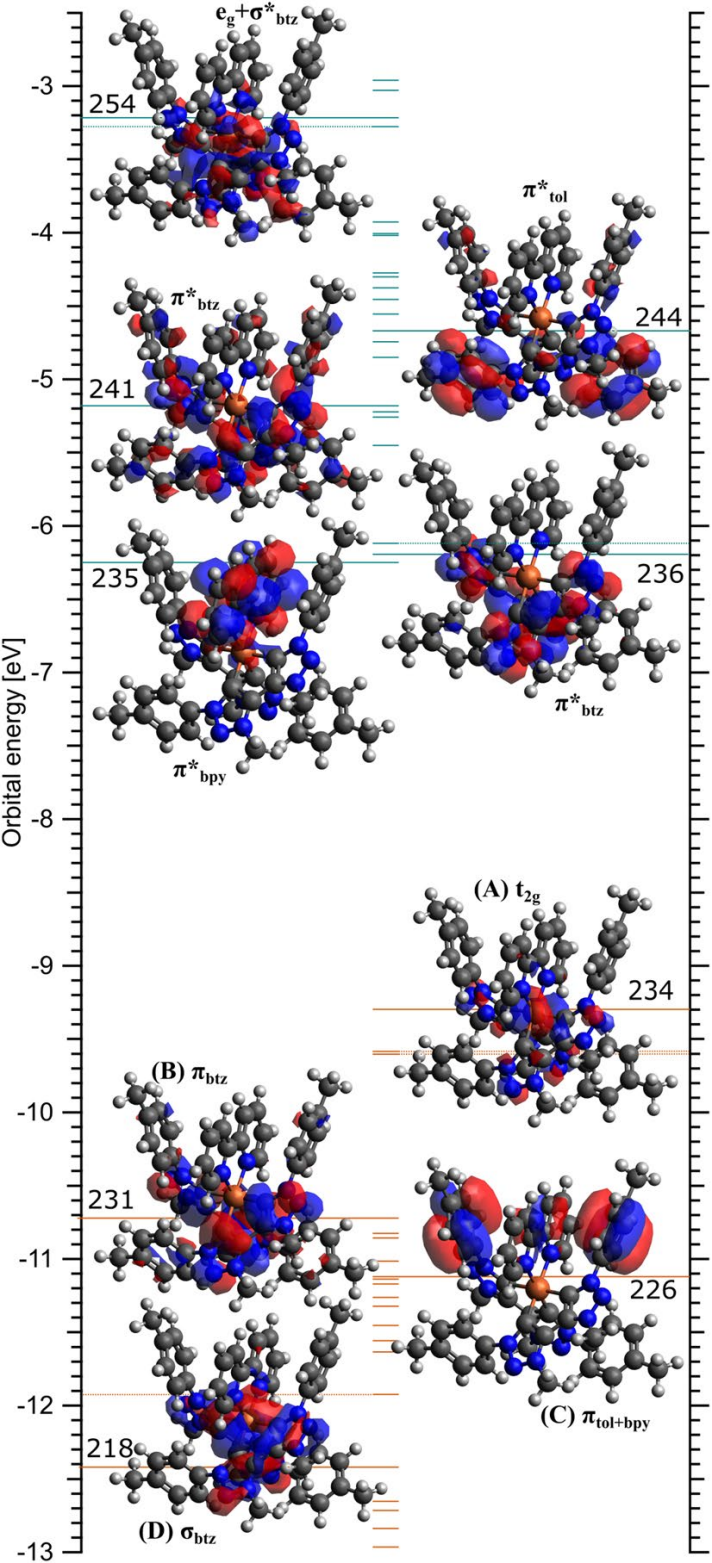
Energies of occupied (brown lines, orbital 234 and below) and unoccupied (turquoise lines, orbitals 235 and above) molecular orbitals (MOs) from ground state density functional theory calculations of an isolated ${[\hbox {Fe}^{{{\rm{II}}}}(\hbox {btz})_2(\hbox {bpy})]^{2+}}$ molecule. Labels (A–D) connect to experimental features in the RPES (see text and Fig. 6). Next to each plotted MO is a description of the dominant character. Dashed lines indicate the orbital is very similar to the nearby MO that is plotted.

Selected ground state frontier molecular orbitals (MOs) of ${[\hbox {Fe}^{{{\rm{II}}}}(\hbox {btz})_2(\hbox {bpy})]^{2+}}$ are presented in Fig. 4 as a basis for the subsequent discussions of the RPES data. Unlike ${[\hbox {Fe}^{{\rm{III}}}(\hbox {btz})_3]^{3+}}$, which we have previously discussed in detail^35^, ${[\hbox {Fe}^{{{\rm{II}}}}(\hbox {btz})_2(\hbox {bpy})]^{2+}}$ is a closed-shell complex. The LUMO (lowest unoccupied MO) is almost entirely of $\pi ^*$ character and located on the bpy ligand (MO-235). At slightly higher energy are a pair of $\pi ^*$ derived orbitals with density predominantly on the btz N atoms (MO-236 and MO-237). The next orbitals, MO-238 and MO-239 (not shown), are $\pi ^*_{bpy}$ and look similar to the LUMO. Next are a series of $\pi ^*$ orbitals on the btz and toluene rings with MO-241 and MO-244 shown as respective examples. Beyond this, MO-253 is the first unoccupied molecular orbital with $\sigma ^*$ and ${\hbox {e}_{{\rm{g}}}}$ character, as are the higher orbitals (MO-254 is a representative example).

**Figure 5:**
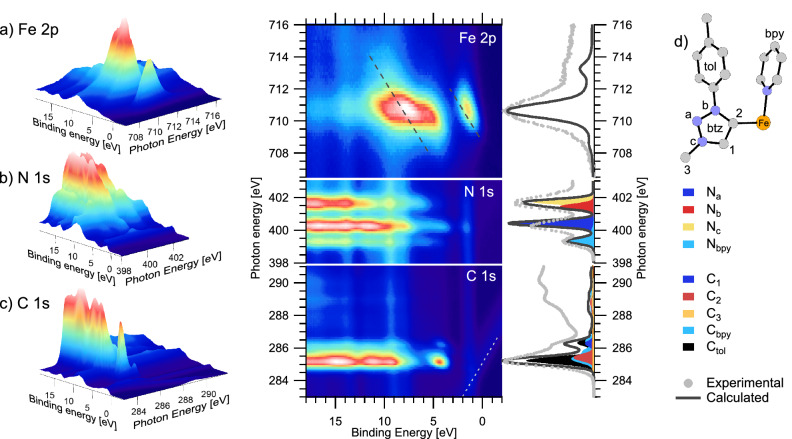
${[\hbox {Fe}^{{{\rm{II}}}}(\hbox {btz})_2(\hbox {bpy})]^{2+}}$ RPES maps showing **(a)** Fe $2p_{3/2}$ (top), **(b)** N 1*s* (middle) and **(c)** C 1*s* (bottom) transitions. Grey dashed diagonal lines on the Fe 2*p* map indicate paths of constant kinetic energy. The white dotted line marks second order C 1*s* photoemission. NEXAFS spectra are presented to the right of the maps. The experimental partial electron yield NEXAFS spectrum is plotted with light grey dots and the calculated NEXAFS spectrum is plotted as dark grey lines. The contribution from the different atoms to the calculated spectra are shown by coloured areas, as detailed in the key and molecule scheme **(d)**.

The HOMO (highest occupied MO) is predominantly of ${\hbox {t}_{{\rm{2g}}}}$ character with a small amount of density on the btz ring, notably on the $\hbox {N}_a$ nitrogen atom (defined in the scheme in Fig. 5d). This was also observed for ${[\hbox {Fe}^{{\rm{III}}}(\hbox {btz})_3]^{3+}}$^35^. The HOMO$-1$ and HOMO$-2$ (not shown) are of similar character. Below this are a series of $\pi$ orbitals localised on the btz, bpy and toluene (tol) moieties. The first of these, MO-231, is localised on the btz rings. The next 10 orbitals (MO-230 to MO-220) are predominantly on the bpy and auxiliary tol groups, where MO-226 is plotted as a representative example. Lower orbitals are of $\sigma$ character, where MO-218 (and MO-219, similar but not shown) show overlap with the central Fe atom. In general, this electronic structure shows the expected characteristics predicted for octahedral ligand-field interactions (Fig. 2) where the results imply mixing of the ${\hbox {t}_{{\rm{2g}}}}$ with $\pi$ orbitals and of the ${\hbox {e}_{{\rm{g}}}}$ with the $\sigma$ orbitals. The ab inito ligand field theory calculations predict a 10Dq (ligand field splitting) of 2.9 eV.

**Figure 6:**
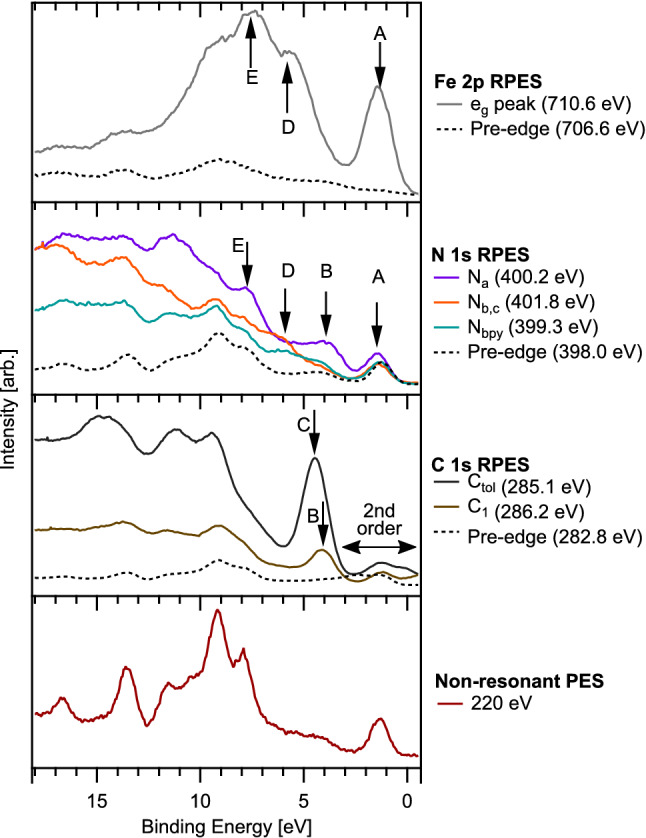
Valence band spectra of ${[\hbox {Fe}^{{{\rm{II}}}}(\hbox {btz})_2(\hbox {bpy})]^{2+}}$, extracted from the RPES maps in Fig. 5 at key resonant transitions. Also included are off-resonance valence band spectra measured in the pre-edge region (dashed lines) and at lower photon energy. The corresponding photon energy of each spectrum is indicated in the key. Features attributed to participant enhancement of frontier molecular orbitals are labelled A–E, and connect to the labelling of the representative molecular orbitals in Fig. 4. The contribution to the C 1*s* RPES originating from direct C 1*s* photoemission from 2nd order light is also labelled.

C 1*s*, N 1*s* and Fe $2p_{3/2}$ RPES measurements of ${[\hbox {Fe}^{{{\rm{II}}}}(\hbox {btz})_2(\hbox {bpy})]^{2+}}$ are presented in Fig. 5 alongside experimental and calculated near edge X-ray absorption fine structure (NEXAFS) spectra. Considering firstly the Fe $2p_{3/2}$ ($\hbox {L}_3$ edge) data, Fig. 5a shows a strong resonance centred around $h\nu \sim 710.5~\hbox {eV}$ corresponding to an Fe $2p_{3/2} \rightarrow {\hbox {e}_{{\rm{g}}}}$ transition. At this photon energy, the RPES map shows strong participant enhancement of the HOMO (the ${\hbox {t}_{{\rm{2g}}}}$-dominated MO-234) at $\sim 1.5\,\hbox {eV}$ binding energy. We note the non-constant binding energy dispersion of the ${\hbox {t}_{{\rm{2g}}}}$ enhancement, which, whilst skewed toward a constant kinetic energy dispersion (diagonal dashed line drawn on map), does not follow this trajectory exactly. Vibronic coupling provides a potential explanation which we explore in the discussion section. The other strong enhancement in the map is between 5 eV and 10 eV binding energy. Whilst this feature contains some participant enhancement, resonant (spectator) enhancement of the Fe LMM Auger signal at $\sim 703\,\hbox {eV}$ (kinetic energy)^47^ also contributes significantly. The feature is elongated along the diagonal, constant kinetic energy direction.

Comparing the N 1*s* RPES measurements of ${[\hbox {Fe}^{{{\rm{II}}}}(\hbox {btz})_2(\hbox {bpy})]^{2+}}$ (Fig. 5b) to previous results of ${[\hbox {Fe}^{{\rm{III}}}(\hbox {btz})_3]^{3+}}$^35^, we note spectral similarities originating from absorption into the carbene ring on the btz ligands centred at $h\nu =~400.2~\hbox {eV}$ and $h\nu =~401.8~\hbox {eV}$. These resonances correspond to excitation from two distinct core levels localised on the $\hbox {N}_a$ and $\hbox {N}_{{b}}$/$\hbox {N}_{{c}}$ atoms (defined in Fig. 5d) into unoccupied $\pi ^*$ molecular orbitals on the btz rings (MO-236 and MO-237 in Fig. 4). The absorption feature at $h\nu =~399.2~\hbox {eV}$ is only present for ${[\hbox {Fe}^{{{\rm{II}}}}(\hbox {btz})_2(\hbox {bpy})]^{2+}}$ and therefore attributed to the bpy ligand. This is consistent with the calculated spectra where this feature corresponds to excitation into $\pi ^*$ MOs on the bpy ligand (MO-235, LUMO). A clear participant enhancement is visible at 1.5 eV binding energy on the $\hbox {N}_a$ resonance ($h\nu =~400.2~\hbox {eV}$). This is the same binding energy as the enhancement of the ${\hbox {t}_{{\rm{2g}}}}$-derived HOMO in the Fe $2p_{3/2}$ RPES map. The enhancement of the HOMO on both the Fe and $\hbox {N}_a$ resonances implies there is mixing of this orbital between the metal centre and btz ligands around the $\hbox {N}_a$ atom. This is consistent with orbital MO-234, which, although dominantly ${\hbox {t}_{{\rm{2g}}}}$ character, shows some density on the $\hbox {N}_a$ atoms (Fig. 4A).

The interpretation of the C 1*s* RPES data is challenging as the spectra include excitations from numerous core-level environments into numerous unoccupied molecular orbitals. This is highlighted by the calculated NEXAFS spectra, Fig. 5c, where we can see the spectral contributions from various carbon environments in the btz, tol and bpy moieties. The main resonance at $h\nu =~285.2~\hbox {eV}$ is dominated by excitation from the C 1*s* orbitals on the toluene ring into a series of $\pi ^*$ orbitals (represented in Fig. 4 by MO-244). Also contributing slightly to the intensity of this feature are equivalent excitations located on the bpy ligands and the “$\hbox {C}_2$” atom (defined in Fig. 5d). The higher photon-energy shoulder has contribution from the tol, bpy and $\hbox {C}_1$ carbon in the btz. Even higher photon-energy structure in the NEXAFS, at photon energies 288 eV - 291 eV, has dominant contributions from $\hbox {C}_1$, $\hbox {C}_2$, $\hbox {C}_3$ and the methyl group in the toluene moiety as shown in more detail in Supplementary Fig. 1. The diagonal feature in the RPES at low binding energy (${+2}\,{\hbox {eV}}$ to ${-2}\,\hbox {eV}$) marked with white dots is C 1*s* photoemission generated by second-order light from the monochromator.

Figure 6 shows individual spectra extracted from the the maps (constant photon energy slices) at key resonances, alongside pre-edge spectra (dashed lines) to aid in distinguishing participant enhancements from direct photoemission of the valence band. A higher-resolution off-resonance spectrum, measured at $h\nu =~220~\hbox {eV}$, is also included for reference. Notable participant enhancements in the RPES maps are labelled A-E. Enhancement “A”, appearing strongly on the Fe ${\hbox {e}_{{\rm{g}}}}$ and slightly on the $\hbox {N}_a$ resonance at ${\sim 1}\,{\hbox {eV}}$ represents the ${\hbox {t}_{{\rm{2g}}}}$ HOMO. The absence of intensity in the C 1*s* RPES implies the ${\hbox {t}_{{\rm{2g}}}}$ is highly localised as predicted by the calculated orbitals (MO-243). Enhancement “B” appears on the $\hbox {N}_a$ spectrum, but also on the “$\hbox {C}_1$” resonance at $h\nu =~286.2~\hbox {eV}$. As shown in the calculated NEXAFS spectrum (Fig. 5), the resonance at $h\nu =~286.2~\hbox {eV}$ shows notable contribution from the $\hbox {C}_1$ atom in the btz rings. This valence feature is well described by MO-231. The strongest enhancement “C” represents dominantly $\pi ^*$ orbitals in the toluene ligands. Beyond this we see enhancements “D” and “E” on the Fe and N, which we attribute to the $\sigma$ orbitals that mix between the btz ligands and the Fe atom (for example MO-218). This direct experimental measurement of the frontier orbital coupling between the $\sigma$ and ${\hbox {e}_{{\rm{g}}}}$ is consonant with the expected ligand-field interactions presented in Fig. 2.

### ${[\hbox {Fe}^{{\rm{III}}}(\hbox {btz})_3]^{3+}}$

**Figure 7:**
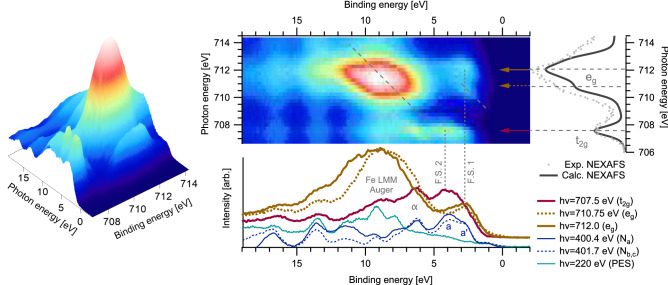
Fe $2p_{3/2}$ RPES and NEXAFS data of ${[\hbox {Fe}^{{\rm{III}}}(\hbox {btz})_3]^{3+}}$. Dashed lines on the maps indicate paths of constant kinetic energy. The NEXAFS spectrum is presented on the right of the map where grey dots show the experimentally measured spectrum (using the partial yield detector) and the solid line the calculated spectrum. Horizontal line profiles have been extracted from the map at the main ${\hbox {t}_{{\rm{2g}}}}$ and ${\hbox {e}_{{\rm{g}}}}$ resonances and plotted with non-resonant PES and N 1*s* RPES data (from Ref.^35^).

Figure 7 presents Fe $2p_{3/2}$ RPES and NEXAFS measurements of ${[\hbox {Fe}^{{\rm{III}}}(\hbox {btz})_3]^{3+}}$. Corresponding N 1*s* and C 1*s* measurements can be found elsewhere^35^. Two distinct absorption features are visible in the data. The first, at $h\nu =~707.5~\hbox {eV}$, is attributed to excitation into the hole in the ${\hbox {t}_{{\rm{2g}}}}$ and the second, 710–713 eV, to transitions into the unoccupied ${\hbox {e}_{{\rm{g}}}}$. The sub-structure of the ${\hbox {e}_{{\rm{g}}}}$ is attributed to a combination of multistate and spin effects similar to what has previously been observed for $[{\hbox {Fe}^{{\rm{III}}}(\hbox {CN})6}]^{3-}$^46^. A calculated NEXAFS spectrum is included to the right of the image plot in Fig. 7; the spectral shape describes the experimental spectrum well.

The energy difference between the ${\hbox {t}_{{\rm{2g}}}}$ and ${\hbox {e}_{{\rm{g}}}}$ in the NEXAFS spectrum provides a measurement of 10Dq, which is in the range of 3.5 to 4.5 eV; the range reflects the internal structure of the ${\hbox {e}_{{\rm{g}}}}$ feature. Our calculations predict a 10Dq of 3.7 eV, which is consistent with the experimental measurement. It is also of comparable strength to the 4.4 eV value reported for the strong sigma donating complex $[{\hbox {Fe}^{{\rm{III}}}(\hbox {CN})6}]^{3-}$^46^.

Spectra were extracted from the RPES map at three excitation energies: the ${\hbox {t}_{{\rm{2g}}}}$ resonance at 707.5 eV and through the two resolvable ${\hbox {e}_{{\rm{g}}}}$ features at 710.8 eV and 712.0 eV. These are plotted below the map in Fig. 7 alongside an off-resonance higher-resolution valence band spectrum, measured at $h\nu =~220~\hbox {eV}$, and two N 1*s* resonant photoemission spectra. The latter were measured at 400.4 eV and 401.7 eV corresponding to the $\hbox {N}_a$ and $\hbox {N}_{b,c}$ environments (a detailed discussion of which can be found elsewhere^35^). These have had the background shape, originating from the resonant enhancement of the N KLL Auger, subtracted to aid comparison to the Fe data.

The intense and broad feature on the ${\hbox {e}_{{\rm{g}}}}$ resonance, between ${\sim 5}\,{\hbox {eV}}$ and ${\sim 15}\,{\hbox {eV}}$ binding energy, is attributed to resonant/spectator enhancement of the Fe LMM Auger. It appears to follow a constant kinetic energy dispersion across the ${\hbox {e}_{{\rm{g}}}}$ resonance, which approximately tracks back to the HOMO enhancement on the ${\hbox {t}_{{\rm{2g}}}}$ resonance. This unique position in the energy landscape theoretically represents a limiting case where participant and spectator decay processes result in an experimentally equivalent final valence configuration of ${(\hbox {t}_{{\rm{2g}}}^4)(\hbox {e}_{\rm{g}}^0)}$. This convergence of spectator and participant decay in RPES is only a possibility in open-shell systems. However, these features on the ${\hbox {t}_{{\rm{2g}}}}$ resonance are well resolved and therefore resemble participant enhancements. The intensity of the Auger feature are shown on a kinetic energy axis in Supplementary Fig. 3 allowing easy comparison of the spectral shapes.

An interesting observation in the Fe $2p_{3/2}$ RPES data is that the lowest binding energy feature in the spectrum measured on the ${\hbox {t}_{{\rm{2g}}}}$ resonance appears around 1 eV higher in energy compared to the spectrum measured on the ${\hbox {e}_{{\rm{g}}}}$ resonance. There is no reason that the participant decay from the two different intermediate states should involve different occupied orbitals. We therefore propose these features, with distinctly separated binding energies, represent participant decay into two different final states. These features are therefore labelled final state (F.S.) 1 and 2, and are further explored in the discussion section.

We note the F.S.1 and F.S.2 enhancements align with features measured in the N 1*s* RPES, labelled $a'$ and *a* in our previous paper^35^. There, feature *a* was attributed to participant enhancement of a $\pi _{btz}$ orbital, whereas the origin of $a'$ was less definitively understood. We also note that whilst this earlier study did contain some limited Fe 2*p* RPES data, the Fe $2p_{3/2}$ spectra presented here dramatically improve on these early measurements. This is largely due to higher-quality uniform films and a better understanding and control of radiation damage (which can cause reduction of the molecule). Repeat measurements allow us to conclude that the limited Fe 2*p* XAS/RPES data presented in reference^35^ likely represents a mix of $\hbox {Fe}^{{\rm{II}}}$ and $\hbox {Fe}^{{\rm{III}}}$ character, whilst there was no such radiation damage apparent in the N 1*s* and C 1*s* data. As elaborated upon in the discussion, we think the F.S.1 enhancement explains the a’ shoulder in the N 1*s* RPES data, but the F.S.2 feature is not the dominant origin of feature a, which we still assign to be mainly $\pi _{btz}$. Coupling between the HOMO on the Fe and N atoms is consistent with both the expected ligand-metal interactions (Fig. 2) and the observations in the ${[\hbox {Fe}^{{{\rm{II}}}}(\hbox {btz})_2(\hbox {bpy})]^{2+}}$ data. Another commonality between the nitrogen and Fe ${\hbox {t}_{{\rm{2g}}}}$ RPES is the enhancement labelled $\alpha$ at ${\sim 6}\,{\hbox {eV}}$. This feature is attributed to MOs containing a mix of bonding Fe ${\hbox {e}_{{\rm{g}}}}$ and $\sigma _{btz}$ contributions.

A resolvable sub-structure is visible in the resonantly enhanced intensity of the HOMO on the ${\hbox {e}_{{\rm{g}}}}$ excitation (F.S.1). Indeed, there appear to be two resonances that occur at ${\sim 710.5}\,{\hbox {eV}}$ and ${\sim 712.0}\,{\hbox {eV}}$. The origins of the two resonances were discussed in the context of the analysis of the NEXAFS spectrum. Further, around the ${\sim 710}\,{\hbox {eV}}$ resonance, the F.S.1 enhancement appears to be skewed towards constant kinetic energy (as highlighted by the dashed line on the RPES map) on the leading edge of the ${\hbox {e}_{{\rm{g}}}}$ XAS resonance. This feature, attributed to vibronic effects, is further explored in the discussion section.

A complementary N 1*s* NEXAFS spectrum is included in Supplementary Fig. 4, with an improved pre-edge region compared to our previously published spectrum^35^. We note the lack of a strong pre-edge feature attributed to N 1*s* $\rightarrow$ Fe 3*d* transitions. This is consistent with our previously published N 1*s* XAS simulation, using TD-DFT, which also does not show any notable pre-edge features corresponding to N 1*s* $\rightarrow$ Fe 3*d* transitions^35^. This type of ligand $\rightarrow$ metal transition is possible in complexes where the ligand np orbitals participate in bonding interactions with the metal atom^48,49^. This has been observed in N 1*s* XAS in open-*d*-shell complexes, such as $[{\hbox {Fe}^{{\rm{III}}}(\hbox {CN})6}]^{3-}$^46^ and ${[\hbox {Ni(NH}{_3}{)}_6]^{2+}}$^48^, but also for other ligand excitations such at the S K-edge^49^. The lack of a pre-edge feature in ${[\hbox {Fe}^{{\rm{III}}}(\hbox {btz})_3]^{3+}}$ is therefore notable given it has a singly unoccupied MO with both metal ${\hbox {t}_{{\rm{2g}}}}$ and ligand $\pi ^*$ character^35^ and the RPES results imply high levels of orbital mixing. A follow-up computational examination of why it manifests strongly in some complexes and not others is underway and will be published in due course.

## Discussion

### Final state propensity

**Figure 8:**
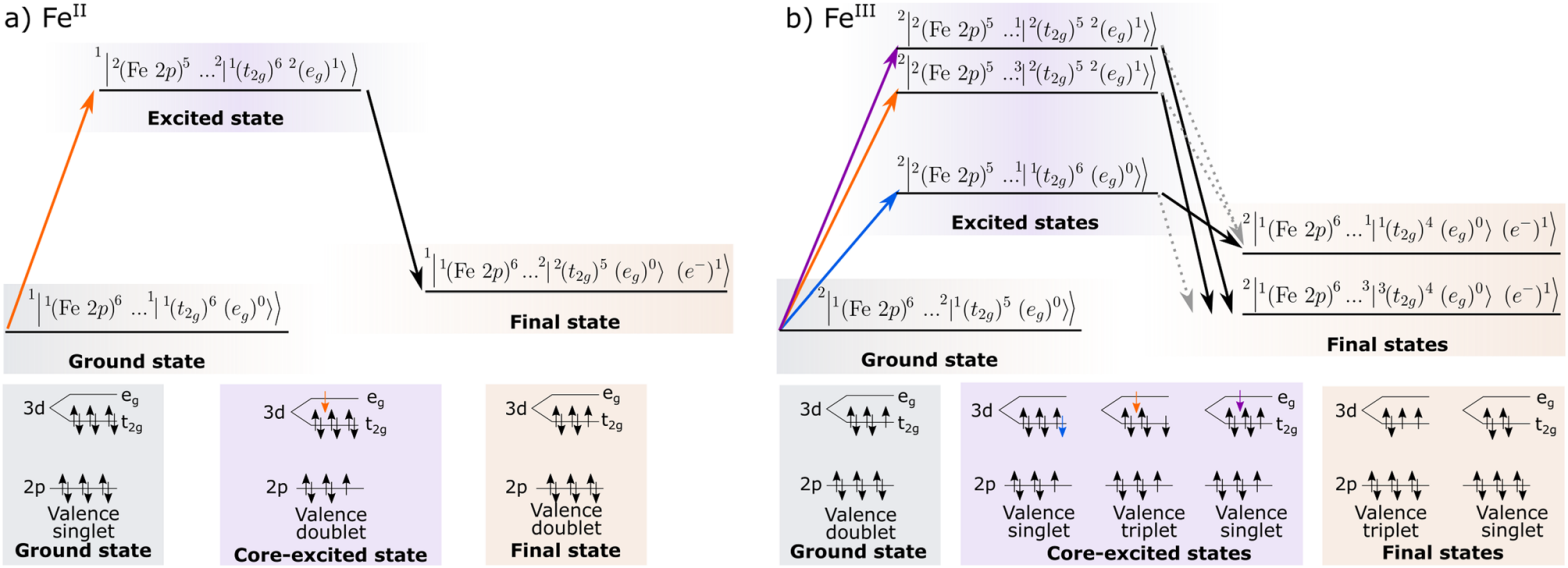
State diagram depicting the relative energies and electron configurations anticipated in Fe L-edge RPES of **(a)** closed-shell $\hbox {Fe}^{{\rm{II}}}$ and **(b)** open-shell $\hbox {Fe}^{{\rm{III}}}$ low spin complexes. In each case the ground/initial state, core-excited intermediate states and final states following participant decay are shown. For each state presented, the core (Fe 2*p*), valence (Fe 3*d* ${\hbox {t}_{{\rm{2g}}}}$ and ${\hbox {e}_{{\rm{g}}}}$ shells) and overall multiplicities are given in the usual convention where 1,2,3 stated before a bar or bracket indicate singlet, doublet and triplet spin configurations. The number following a ket or bracket indicates the occupancy. ($\hbox {e}^-$) is used to denote the emitted photoelectron. They grey dashed arrows indicate suppressed transitions.

The process of an Fe 2*p* resonant excitation followed by participant decay involving the ${\hbox {t}_{{\rm{2g}}}}$ HOMO can be described in the manifold of ground, intermediate (core-excited) and final (valence-ionised) states as recently described for the $\hbox {Fe}^{{\rm{II}}}$ and $\hbox {Fe}^{{\rm{III}}}$ hexacyanide model complexes^43^. Figure 8 provides a simplified scheme considering the relevant metal-centred states. Any complexities originating from hybridisation effects, shakeup excitations etc. are ignored. It is worth noting that there are several singlet and triplet states with different populations of the ${\hbox {t}_{{\rm{2g}}}}$ levels (see Supplementary Figs. 5 and 6), but they are sufficiently close in energy such that they are not experimentally resolvable.

In the $\hbox {Fe}^{{\rm{II}}}$ case (Fig. 8a) the state picture describing participant enhancement of the ${\hbox {t}_{{\rm{2g}}}}$ is simple. There is one possible resonant transition localised on the Fe atom where an electron is excited from the 2*p* level to the ${\hbox {e}_{{\rm{g}}}}$ producing a valence doublet in the core-excited state. From here, there is only one possible participant decay channel which results in a valence doublet final state.

Participant enhancement in the open-shell $\hbox {Fe}^{{\rm{III}}}$ case (Fig. 8b) is more complicated as Fe 2*p* RPES can lead to multiple core-excited intermediate states and multiple final states. Firstly, considering the core-excited states, excitation into both the ${\hbox {t}_{{\rm{2g}}}}$ and ${\hbox {e}_{{\rm{g}}}}$ orbitals is possible. The excitation into the ${\hbox {t}_{{\rm{2g}}}}$ hole completes the ${\hbox {t}_{{\rm{2g}}}}$ shell so there is only one possible valence electronic configuration in the core-excited state: a valence singlet, i.e. $^1|(t_{2g})^6\rangle$. When exciting into the ${\hbox {e}_{{\rm{g}}}}$ both overall valence singlet, $^1|(t_{2g})^5~(e_g)^1\rangle$, and valence triplet, $^3|(t_{2g})^5~(e_g)^1\rangle$, intermediate states are possible. After participant decay, the final state of the complex can either be a valence singlet $^1|(t_{2g})^4\rangle$ or valence triplet $^3|(t_{2g})^4\rangle$ depending on the spin of the ${\hbox {t}_{{\rm{2g}}}}$ electrons that participate.

Quantum chemical calculations of ${[\hbox {Fe}^{{\rm{III}}}(\hbox {btz})_3]^{3+}}$ predict the lowest-energy singlet to be 1.6 eV above the lowest-energy triplet final state (details in Supplementary Fig. 6). Experimentally, we observe the participant enhancement of the ${\hbox {t}_{{\rm{2g}}}}$ HOMO at ${\sim 2.5}\,{\hbox {eV}}$ (labelled F.S. 1) and ${\sim 4.0}\,{\hbox {eV}}$ (F.S. 2) at the ${\hbox {e}_{{\rm{g}}}}$ and ${\hbox {t}_{{\rm{2g}}}}$ resonances, respectively. This 1.5 eV splitting between the experimental features is consistent with the computational result. The experiment suggests that the ${\hbox {e}_{{\rm{g}}}}$ excitation dominantly leads to the lower-energy triplet final state, which is consistent with simple spin statistics the triplet state has three $m_s$ components while the singlet state has only one. In contrast, the ${\hbox {t}_{{\rm{2g}}}}$ excitation seemingly leads dominantly to the higher energy singlet configuration. This is a manifestation of spin propensity, where transitions involving a spin-up and spin-down pair of electrons are favoured over transition involving pairs with aligned spins. This selection rule has previously been observed in small gas-phase molecules, including $\hbox {NO}_2$^50^ and $\hbox {O}_2$^51^, and recently in iron hexaferricyanide^43^. Additionally, Auger spectroscopy measurements of Kr have shown a preference towards singlet coupling for some of the LMM transitions^52^. Applying this propensity rule to ${[\hbox {Fe}^{{\rm{III}}}(\hbox {btz})_3]^{3+}}$ leads to restrictive spin transitions following the ${\hbox {t}_{{\rm{2g}}}}$ excitation: the valence triplet final state is suppressed as the de-excitation requires the two participating electrons to have the same spin.

### Oxidation state effects

As the function of transition metal complexes is intimately coupled to their versatile oxidation states, it is interesting to note that RPES probes the oxidised final states on the binding energy scale. RPES can therefore expose what can be termed resonant photo-oxidation properties: a view of how electronic structure properties of consecutive oxidation states connect. This provides a snapshot of unrelaxed oxidised species that is different from what can be readily probed spectroscopically on chemically or electrochemically oxidised species. As a step in this direction, we here compare the RPES features the $\hbox {Fe}^{{\rm{II}}}$ $\rightarrow$ $\hbox {Fe}^{{\rm{III}}}$ and $\hbox {Fe}^{{\rm{III}}}$ $\rightarrow$ $\hbox {Fe}^{{\rm{IV}}}$ resonant photo-oxidation processes.

**Figure 9:**
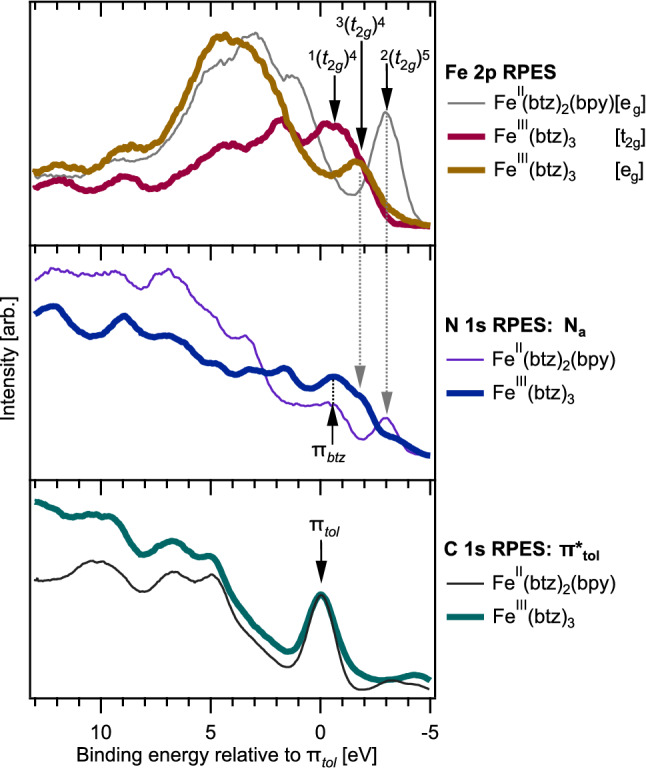
Comparison of ${[\hbox {Fe}^{{{\rm{II}}}}(\hbox {btz})_2(\hbox {bpy})]^{2+}}$ (thin lines) and ${[\hbox {Fe}^{{\rm{III}}}(\hbox {btz})_3]^{3+}}$ (bold lines) RPES at X-ray transitions that the two complexes have in common. The spectra are calibrated relative to the participant enhancement of the $\pi _{tol}$ orbital to allow peak positions to be directly compared between the two complexes.

Figure 9 collates the resonant photoemission spectra, on the Fe, $\hbox {N}_a$ and C (toluene-dominated) $\pi ^*$ resonances, that are common between ${[\hbox {Fe}^{{{\rm{II}}}}(\hbox {btz})_2(\hbox {bpy})]^{2+}}$ and ${[\hbox {Fe}^{{\rm{III}}}(\hbox {btz})_3]^{3+}}$. These spectra are calibrated relative to the $\pi _{tol}$ participant enhancement (set to 0 eV). The N and Fe spectra were shifted accordingly, maintaining the relative energy positions for each molecule. As the toluene moiety is physically distant from the central Fe atom, the effect of Fe oxidation state on the toluene atoms is likely negligible. The $\pi _{tol}$ peak therefore provides a compelling binding energy reference, allowing a comparison of the two samples and negating experimental factors that can effect the measured photoelectron energies. Figure 9 also acts to summarise our assignments of key RPES features.

First, we note the spectral similarity observed in the C 1*s* $\pi ^*_{tol}$ RPES of the two molecules. The similarity is evidence that the bpy ligand in ${[\hbox {Fe}^{{{\rm{II}}}}(\hbox {btz})_2(\hbox {bpy})]^{2+}}$ does not make a significant contribution to this particular spectrum/resonance. The common shape for both the $\hbox {Fe}^{{\rm{II}}}$ and $\hbox {Fe}^{{\rm{III}}}$ spectra therefore validates the resonances are $\pi ^*_{tol}$ dominated and thus the calibration approach.

Considering the Fe $2p_{3/2}$ spectra, the feature attributed to the ${\hbox {t}_{{\rm{2g}}}}$-derived HOMO for ${[\hbox {Fe}^{{{\rm{II}}}}(\hbox {btz})_2(\hbox {bpy})]^{2+}}$ lies ${\sim 1.3}\,{\hbox {eV}}$ above that of ${[\hbox {Fe}^{{\rm{III}}}(\hbox {btz})_3]^{3+}}$. This type of binding energy difference is expected given the difference in oxidation state and thus the charge on the iron atom. This difference is of a comparable size to that between $[{\hbox {Fe}^{{\rm{II}}}(\hbox {CN})_6]^{4-}}$ and $[{\hbox {Fe}^{{\rm{III}}}(\hbox {CN})_6]^{3-}}$, which has been measured as 1.3 eV^43^ and 1.4 eV^53^. This binding energy shift of the HOMO feature is also potentially explained, in part, by differing levels of $\pi ^*$ back-donation, which acts to stabilise the ${\hbox {t}_{{\rm{2g}}}}$, i.e. shifting it to higher binding energy.

It is interesting that the spin-resolved final state features assigned to autoionisation of the ${\hbox {t}_{{\rm{2g}}}}$ orbital appear in both the Fe and N RPES data. This is particularly apparent for the $^2(t_{2g})^5$ state of ${[\hbox {Fe}^{{{\rm{II}}}}(\hbox {btz})_2(\hbox {bpy})]^{2+}}$ and the $^3(t_{2g})^4$ state of ${[\hbox {Fe}^{{\rm{III}}}(\hbox {btz})_3]^{3+}}$ (the co-existance of these features are marked with dashed arrows in Fig. 9). Such strong enhancements of these metal states in the nitrogen data implies very strong mixing of the ${\hbox {t}_{{\rm{2g}}}}$ and $\pi _{btz}$ (depicted in Fig. 2 as a $\pi$ donation interaction). This is in clear contrast to the lack of a pre-edge feature in the N 1*s* X-ray absorption spectrum, which would imply the metal does not extensivly mix with the ligand $\pi ^*$ orbitals ($\pi ^*$ back- donation interactions in Fig. 2).

We are unable to definitively state whether the $^1(t_{2g})^4$ final state of ${[\hbox {Fe}^{{\rm{III}}}(\hbox {btz})_3]^{3+}}$ also appears in the N 1*s* resonant photoemission spectra as this feature would overlap with the enhancement of orbitals that are dominantly of $\pi _{btz}$ with negligible metal character. The assignment of this feature as $\pi _{btz}$ is based on the intensity of the enhancement in the RPES and the calculated orbital energies as discussed elsewhere^35^. It is also interesting that this feature aligns perfectly with the equivalent feature in ${[\hbox {Fe}^{{{\rm{II}}}}(\hbox {btz})_2(\hbox {bpy})]^{2+}}$, which implies the coulombic difference between the two oxidation state has little influence over the dominantly $\pi _{btz}$ orbitals that participate in this resonant enhancement.

### Vibronic coupling

**Figure 10:**
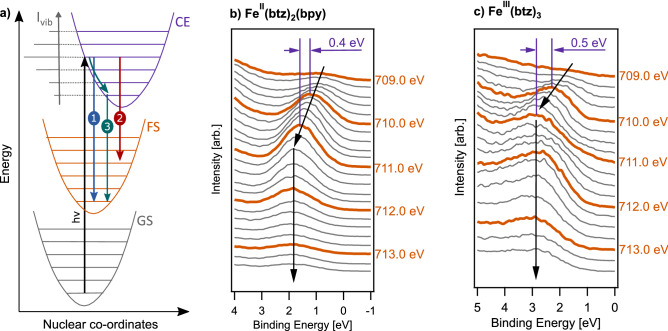
Scheme and evidence of vibronic coupling observed in the Fe $2p_{3/2}$ RPES data of both FeNHC complexes **(a)** Simplified Franck–Condon diagram highlighting the different relaxation possibilities after excitation into higher vibrational modes in the core-excited intermediate state. *GS* ground (initial) state, *CE* core excited intermediate state, *FS* final state. **(b**, **c)** show the binding energy evolution of the ${\hbox {t}_{{\rm{2g}}}}$ derived HOMO as a function of photon energy over the Fe $2p_{3/2}$ $\rightarrow$ ${\hbox {e}_{{\rm{g}}}}$ resonance for ${[\hbox {Fe}^{{{\rm{II}}}}(\hbox {btz})_2(\hbox {bpy})]^{2+}}$ and ${[\hbox {Fe}^{{\rm{III}}}(\hbox {btz})_3]^{3+}}$ respectively.

As mentioned, for both ${[\hbox {Fe}^{{{\rm{II}}}}(\hbox {btz})_2(\hbox {bpy})]^{2+}}$ and ${[\hbox {Fe}^{{\rm{III}}}(\hbox {btz})_3]^{3+}}$ we observe participant enhancement of the ${\hbox {t}_{{\rm{2g}}}}$ HOMO in the Fe $2p_{3/2}$ RPES that does not follow a constant binding energy dispersion. Constant kinetic energy dispersion of participant features has previously been ascribed to vibronic coupling effects. This is explored in Fig. 10a, where the local potential energy surfaces, at the location of the core hole, are shown schematically. Here, after resonant excitation into a higher vibrational state, three alternative decay mechanisms are illustrated: decay to the lowest energy final state (1), decay into a vibrationally hot final state (2), and dissipation of the vibrational wave packet away from the core-hole site followed by decay from the lowest vibrational state (of the core-excited state) into the final state (3). When discussing these mechanism in the context of RPES is important to remember that the core hole is localised, and thus not sensitive to long-range effects, and that the core-hole lifetime is short ( ${\sim 1.6}\,{\hbox {fs}}$, determined from the “recommended” Fe $\hbox {L}_3$ natural linewidth of 0.41 eV^54^).

Similar vibrational losses have previously been described for resonant core excitations to anti-bonding levels of small molecules such as $\hbox {BCl}_3$ and $\hbox {CO}_2$^55,56^, and also in larger molecular systems like $\hbox {C}_{60}$ and bi-isonicotinic acid^57,58^. The explanations for the energy dispersion features depend on the size of the molecular system: For $\hbox {CO}_2$, the description is in terms of the transfer of local vibrational energy to the final state (mechanism 2 in Fig. 10a)^56^, whereas for $\hbox {C}_{60}$ and bi-isonicotinic acid, the observation is explained as an ultrafast dissipation of the vibrational wave packet away from the core-hole site. This implies that the decay takes place from the vibrational ground state of the core-excited state (mechanism 3 in Fig. 10a)^57,58^. Another important consideration for high-symmetry molecules featuring pseudo-Jahn-Teller effects is the vibronic coupling in the core-excited state, which has previously been discussed in detail for $\hbox {BCl}_3$^55^.

Figure 10 also shows how the participant enhancement of the ${\hbox {t}_{{\rm{2g}}}}$ HOMO evolves as a function of photon energy, over the ${\hbox {e}_{{\rm{g}}}}$ resonance, for ${[\hbox {Fe}^{{{\rm{II}}}}(\hbox {btz})_2(\hbox {bpy})]^{2+}}$ and ${[\hbox {Fe}^{{\rm{III}}}(\hbox {btz})_3]^{3+}}$. In both cases, an intermediate energy-dispersion profile between that of constant binding and kinetic energies (${\sim 0.5}\,{\hbox {eV}}$ BE per 1 eV $h\nu$) is clearly observable in the photon energy range ${\sim 709}\,{\hbox {eV}}$ to ${\sim 711}\,{\hbox {eV}}$. Peak fitting of this HOMO feature, in the spectra measured at 710.0 eV and 711.0 eV photon energy, quantifies the dispersion over this 1 eV photon energy range to be $0.42\pm 0.03 \,\hbox {eV}$ for ${[\hbox {Fe}^{{{\rm{II}}}}(\hbox {btz})_2(\hbox {bpy})]^{2+}}$ and $0.56\pm 0.28 \,\hbox {eV}$ for ${[\hbox {Fe}^{{\rm{III}}}(\hbox {btz})_3]^{3+}}$. This is detailed in Supplementary Fig. 7 and Supplementary Table 2. Also in both cases, this intermediate dispersion occurs over the first ${\sim 1.5}\,{\hbox {eV}}$ photon energy of the ${\hbox {e}_{{\rm{g}}}}$ resonance, after which the feature follows a conventional constant binding energy trajectory. Interestingly, this intermediate dispersion profile is different to the constant kinetic energy behaviour observed in both the small and large cases described above. Our observations therefore can only be reconciled from an energy-conservation point of view if it is assumed that this intermediate dispersion corresponds to a partial transfer of the vibrational energy through the course of the RPES process.

The vibrational broadening of X-ray absorption features can be associated with significantly displaced core-excited potential energy surfaces relative to the molecular ground state geometry. This is illustrated in Fig. 10a by the shift in nuclear co-ordinates of the core-excited state. In the case of the iron–carbene complexes, this fits well with the established view of elongation of Fe–C bonds through population in the core-excited state of the anti-bonding ${\hbox {e}_{{\rm{g}}}}$ level^8^. This is similar to a mechanism used to explain the vibrational structure of core excitations in small molecules such as $\hbox {CO}_2$^56^. Additionally, bond elongation in the iron carbenes is known to cause symmetry-breaking Jahn–Teller distortions in the metal-centred excited states^8^. This is reminiscent of the vibronic coupling model in the pseudo-Jahn–Teller case of $\hbox {BCl}_3$^55^.

Recent optical pump/X-ray probe measurements of a related iron-carbene complex indicate that vibrations of the Fe–Ligand bonds dissipate on the picosecond timescale^34^. While it needs to be kept in mind that the optical exciation, in principle, has a much larger spatial extent than the X-ray one, it is nevertheless tempting to infer a parallel between the optical and X-ray experiment. Additionally, the Jahn-Teller distortions of the Fe–C bonds promoted by the ${\hbox {e}_{{\rm{g}}}}$ population are expected to be relatively localised in comparison to the delocalised vibrational modes expected in other large molecular cases such as $\hbox {C}_{60}$^57^. Thus it is possible that the energy dispersion we observe in the RPES maps is a fingerprint of de-excitation into vibrationally hot final states where the vibrations dissipate over a much longer timescale than the core-hole lifetime. However, differences in the decoupling and eventual dissipation of the vibrational energy in the core-excited and final states cannot be distinguished from our current experiments alone. Additionally, recent RPES measurements of aqueous solutions of $\hbox {Fe}^{{\rm{II}}}$ and $\hbox {Fe}^{{\rm{III}}}$ hexacyanide do not show any evidence of vibrational losses^43^. Although one would expect these model complexes to behave in a similar way to ${[\hbox {Fe}^{{{\rm{II}}}}(\hbox {btz})_2(\hbox {bpy})]^{2+}}$ and ${[\hbox {Fe}^{{\rm{III}}}(\hbox {btz})_3]^{3+}}$, it is worth noting that not only is the ligand environment different but also the Fe hexacyanides were measured in aqueous solutions. But regardless, it is clear that both the intermediate dispersion and the vibronic decoupling dynamics, over the core-excitation lifetime, warrant further investigation.

## Conclusions

The frontier electronic structure of the $\hbox {Fe}^{{\rm{II}}}$ and $\hbox {Fe}^{{\rm{III}}}$ *N*-heterocyclic carbene complexes ${[\hbox {Fe}^{{{\rm{II}}}}(\hbox {btz})_2(\hbox {bpy})]^{2+}}$ and ${[\hbox {Fe}^{{\rm{III}}}(\hbox {btz})_3]^{3+}}$ were analysed using RPES and supporting quantum chemistry calculations. The participant enhancement of the resonantly-oxidised final states proved to be a sensitive probe of spin-states, ligand-field interactions and ultra-fast vibrational effects that are directly relevant to the functionality of the complexes. The measurements also enabled a comparison of the oxidation state and the closed- vs open- shell nature of the complexes.

RPES measurements of the open-shell ${[\hbox {Fe}^{{\rm{III}}}(\hbox {btz})_3]^{3+}}$ provided access to both ${^1(\hbox {t}_{{\rm{2g}}})^4}$ and ${^3(\hbox {t}_{{\rm{2g}}})^4}$ metal-centred final states, which have an energy separation of 1.5 eV. Final state spin propensity is also present in the data, where the choice in the resonant transition leads to selection of the dominant spin-state. Specifically, the ${\hbox {t}_{{\rm{2g}}}}$ excitation is shown to have a dramatic propensity towards the higher-energy singlet electron configuration compared to the triplet state. This extends upon studies of model complexes^43^ by demonstrating applicability of this approach in a real photofunctional system. The participant enhancement of the ${\hbox {t}_{{\rm{2g}}}}$ final states in both complexes also shows an intriguing energy loss, occurring on the femtosecond timescale, alike that of vibronic coupling. The specific energy dispersion observed points towards the complexes being left in a vibrationally hot final state after resonant core-excitation.

This study provided direct experimental evidence of a high degree of mixing in the frontier metal 3*d* and ligand $\pi _{btz}$ occupied molecular orbitals whilst showing significantly less mixing of the unoccupied orbitals. Additionally, the binding energy of the $\pi _{btz}$ dominated states do not appear to be affected by the Fe oxidation state. For the case of ${[\hbox {Fe}^{{{\rm{II}}}}(\hbox {btz})_2(\hbox {bpy})]^{2+}}$, the RPES data shows no evidence of strong orbital mixing between the bpy ligand and the Fe centre. The 10Dq value was calculated to be 2.9 eV and 3.7 eV for ${[\hbox {Fe}^{{{\rm{II}}}}(\hbox {btz})_2(\hbox {bpy})]^{2+}}$ and ${[\hbox {Fe}^{{\rm{III}}}(\hbox {btz})_3]^{3+}}$ respectively. Measurements of ${[\hbox {Fe}^{{\rm{III}}}(\hbox {btz})_3]^{3+}}$ provide a measurement of the 10Dq in the range 3.5 to 4.5 eV, consistent with the calculated value.

The RPES maps presented here provide a richer understanding of the Fe NHC complexes, from the perspective of both the metal and ligand, whose photofunctionality relies on excited state ligand-metal charge transfer interactions. Our observation of high levels of $\pi$-${\hbox {t}_{{\rm{2g}}}}$ mixing, where the photo-oxidized metal centred state is also accessible from the ligand, provides site-specific information about the electronic structure that complements the understanding from the optical LMCT excitations in the $\hbox {Fe}^{{\rm{III}}}$ system. It also highlights the importance of the ${\hbox {t}_{{\rm{2g}}}}$-$\pi _{btz}$ mixing as part of the ligand design strategy for tuning the photoelectrochemical properties of the iron carbene complexes in general, and for the LMCT properties of the $\hbox {Fe}^{{\rm{III}}}$ carbene complexes in particular. Additionally, the ability of RPES to probe incrementally higher oxidation states is relevant to the application of Fe NHC complexes in (photo)electrochemical reaction cycles where the redox influence on the electronic structure properties is critical. Finally, understanding energy losses through internal vibrations remains of importance to maximising the efficiency of any light harvester. The above provides a compelling justification for future studies of coordination complexes using RPES where steady state, time-resolved and in-operando measurements have the potential to provide a detailed and operational understanding of photochemical systems.

## Methods

### Sample preparation

The FeNHC complexes ${[\hbox {Fe}^{{{\rm{II}}}}(\hbox {btz})_2(\hbox {bpy})]^{2+}}$ and ${[\hbox {Fe}^{{\rm{III}}}(\hbox {btz})_3]^{3+}}$ (schemes in Fig. 1) were prepared following literature protocols^14,19^. Molecular thin films (${\sim 5}\,{\hbox {nm}}$) were prepared by were prepared by spin casting, from acetonitrile solutions, onto gold substrates (Au(111) on mica, from *Georg Albert PVD*) in a nitrogen atmosphere glove box.

### X-ray spectroscopy

The samples were transferred quickly from the glovebox through air into vacuum (${\sim 1\times 10^{-9}}\,{\hbox {mbar}}$) at the HE-SGM beamline/endstation at the Helmholtz-Zentrum Berlin BESSY II synchrotron facility. This bending magnet beamline provides a ${\sim 1}\,{\hbox {mm}} \times {\sim 0.2}\,{\hbox {mm}}$ X-ray spot on the sample with photons in the energy range 200 eV − 800 eV. The measurement chamber was equipped with a Scienta R3000 hemispherical analyser for X-ray photoelectron spectroscopy and a partial electron yield detector for near edge X-ray absorption fine structure (NEXAFS) spectroscopy. All measurements were taken at room temperature.

C and N K-edge NEXAFS was measured using the partial electron yield detector with a 150 V retardation potential. The Fe $2p_{3/2}$ $\hbox {L}_3$-edge NEXAFS was measured with a 470 V retardation potential. A linear background, fitted to the pre-edge background, was subtracted from the NEXAFS spectra. Binding energy scales were calibrated to the Au 4*f*$_{7/2}$ peak of the underlying Au substrate, measured to be at 83.9 eV referenced to the Fermi edge of a clean substrate. RPES measurements were conducted in fixed mode. Higher-resolution, non-resonant PES was measured in swept mode. Approximate beamline and analyser resolutions are listed in Supplementary Table 1. The photon energy was calibrated at the C and N K-edge by measuring the kinetic energy difference between photoemission peaks generated from first- and second-order light from the monochromator. This was not possible for the Fe $\hbox {L}_3$-edge measurements, which was referenced to the nearby F K-edge, with the signal originating from the $\hbox {PF}_6$ counterions, providing a relative but not absolute energy calibration.

Radiation damage from X-ray exposure was monitored using Fe $\hbox {L}_3$-edge NEXAFS spectroscopy and mitigated by regularly moving the sample at appropriate intervals. Notable radiation damage only transpired to be a major concern for resonant excitation of the Fe atom in ${[\hbox {Fe}^{{\rm{III}}}(\hbox {btz})_3]^{3+}}$. In this case, after extended exposure, the NEXAFS spectrum showed partial reduction from $\hbox {Fe}^{{\rm{III}}}$ to $\hbox {Fe}^{{\rm{II}}}$. This was mitigated for the Fe $2p_{3/2}$ RPES map by moving the sample at each new photon energy such that every horizontal line in the map was measured on a fresh area of sample—no damage was observed after this amount of exposure.

### Computational

The geometries used in the X-ray absorption spectra calculations are from density functional theory (DFT) optimisations, which have been published previously^14,19^. Calculations used the ORCA software package^59^. The nitrogen and carbon K-edge NEXAFS was simulated using time-dependent density functional theory (TD-DFT) and the DFT/ROCIS method^60^ respectively. The B3LYP functional and the 6-31G(d) basis set were used. The resulting spectra were broadened with a Gaussian function with a full width at half maximum (FWHM) of 0.4 eV to match the instrumental resolution.

The iron $\hbox {L}_3$-edge XAS was calculated using the restricted active space (RAS) approach with ANO-RCC-VDZ basis set in OpenMolcas^61^. An active space of three Fe 2*p* core orbitals (RAS1), five Fe 3d character orbitals together with two ${\sigma }$ donating orbitals and three empty ${\pi ^*}$ orbitals (RAS2) was used. This method has been successfully used to calculate the L-edge NEXAFS for other 3d transition metal complexes^31,46,62–65^. For comparison to the experimental spectra, the simulated Fe L-edge x-ray abdsorption spectra are plotted using a Lorentzian broadening with a FWHM of 0.2 eV and a Gaussian broadening of 0.3 eV. The calculated spectra were shifted in energy to align with the experimental measurements.

The ab inito ligand field theory (AILFT) calculations were performed by using the complete active space (CAS) SCF method on an *n* electrons ($\textit{n}= 5$, 6), five orbitals (*n*,5) active space. The CASSCF calculations were followed by an N-electron valence ${2^{{\rm{nd}}}}$ order perturbation treatment of dynamic correlation. The full sets of states with a total of 50 singlets, 45 triplets and 5 quintets were calculated for $3\hbox {d}^6$ complex (${[\hbox {Fe}^{\rm{II}}\hbox {(bpy)(btz)}_2]^{2+}}$). The $3\hbox {d}^5$ complex (${[\hbox {Fe}^{{\rm{III}}}(\hbox {btz})_3]^{3+}}$) with a (5, 5) active space spans a total of 75 doublets, 24 quartets and 1 sextet, which were all included in the calculation. The Racah parameters B and C parameters are available in the output, which can be used together with the valence excitation energies and Tanabe-Sugano diagram to evaluate the ligand-field splitting (10Dq).

## Supplementary Information

Supplementary Information.
