# Supplementary material for: Resonant X-ray photo-oxidation of light-harvesting iron (II/III) N-heterocyclic carbene complexes
Source: Sci Rep. 2021 Nov 12;11:22144. doi: 10.1038/s41598-021-01509-7 (PMC8590020; doi:10.1038/s41598-021-01509-7)
Supplement: Supplementary file 1 — Supplementary Information. [file 41598_2021_1509_MOESM1_ESM.pdf]

Supplementary Information: Resonant X-ray photo-oxidation of light-harvesting iron (II/III) *N*-heterocyclic carbene complexes

Robert H. Temperton[1,2,3], Meiyuan Guo[4], Giulio D'Acunto[5], Niclas Johansson[5], Nils W. Rosemann[4], Om Prakash[6], Kenneth Wärnmark[6], Joachim Schnadt[1,3,5,*], Jens Uhlig[3,4,*], and Petter Persson[3,7,*]

[1]MAX IV Laboratory, Lund University, Box 118, 221 00 Lund, Sweden.
[2]School of Physics and Astronomy, University of Nottingham, Nottingham NG7 2RD, U.K.
[3]Lund Institute of Advanced Neutron and X-ray Science, IDEON Building: Delta 5, Scheelevägen 19, 223 70 Lund, Sweden.
[4]Division of Chemical Physics, Department of Chemistry, Lund University, Box 124, 221 00 Lund, Sweden.
[5]Division of Synchrotron Radiation Research, Department of Physics, Lund University, Box 118, 22 100 Lund, Sweden.
[6]Centre for Analysis and Synthesis, Department of Chemistry, Lund University, Box 124, 221 00 Lund, Sweden.
[7]Division of Theoretical Chemistry, Department of Chemistry, Lund University, Box 124, 221 00 Lund, Sweden.
[*]petter.persson@teokem.lu.se, jens.uhlig@chemphys.lu.se, joachim.schnadt@sljus.lu.se

Supplementary Table 1. Estimated resolutions of the beamline and electron analyser at the settings used in the experiments.

Photon Energy [eV]	Beamline Resolution [eV]	Analyser Pass Energy [eV]	Analyser Resolution [eV]	Total Resolution [eV]
710	0.4	200	0.59	0.71
400	0.2	200	0.59	0.63
280	0.1	200	0.59	0.60
220	0.1	100	0.29	0.31

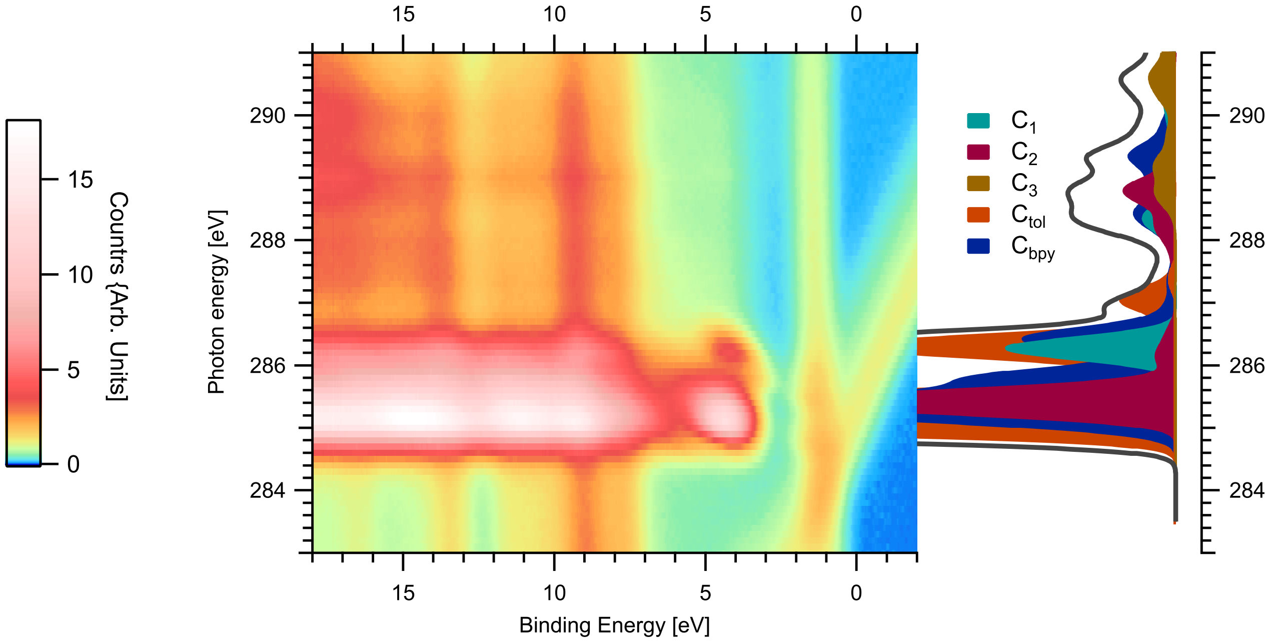

Supplementary Figure 1. $[Fe^{II}(btz)_2(bpy)]^{2+}$ C 1*s* RPES map and calculated NEXAFS spectrum zoomed in to see detail in weak resonant features. For definitions of different carbon contributions, see main manuscript.

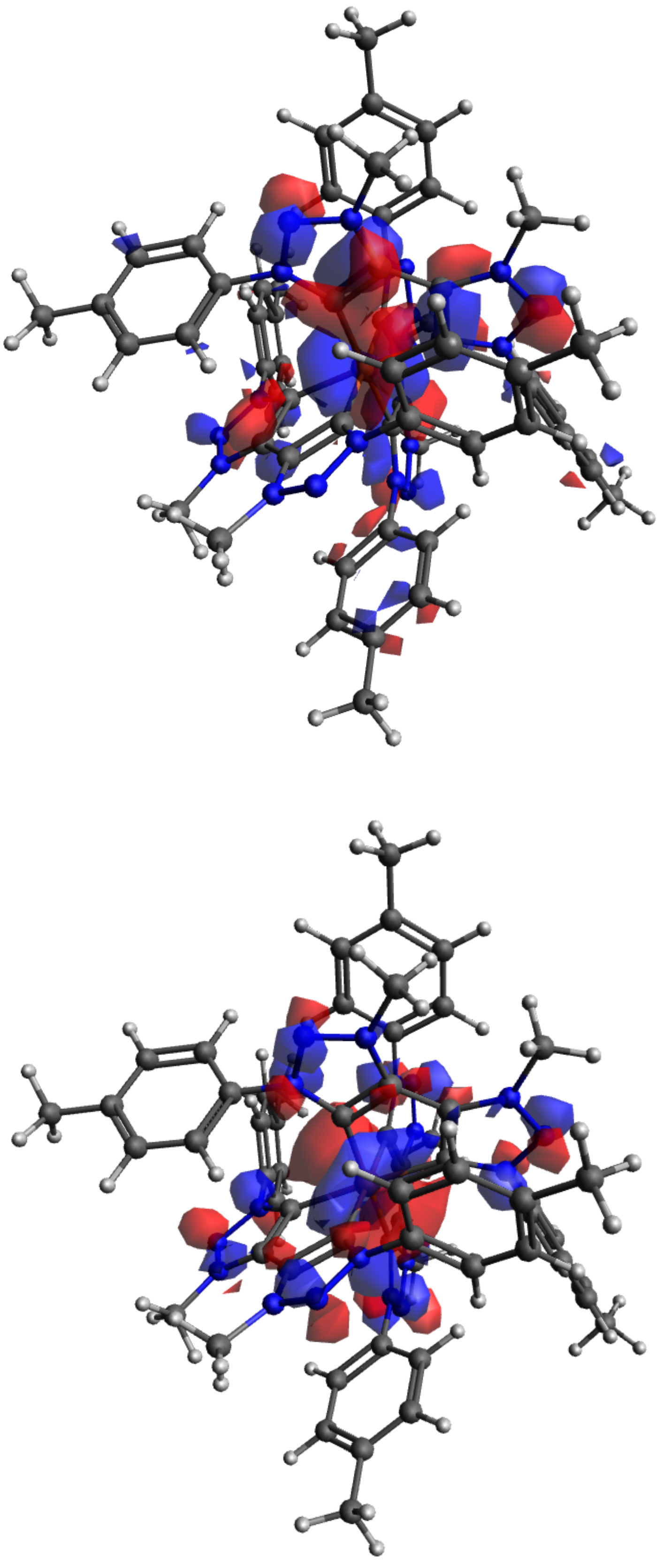

Supplementary Figure 2. $[Fe^{III}(btz)_3]^{3+}$ frontier t_{2g}-derived orbitals. Top: $\beta-283$ (HOMO). Bottom $\beta-284$ (SUMO).

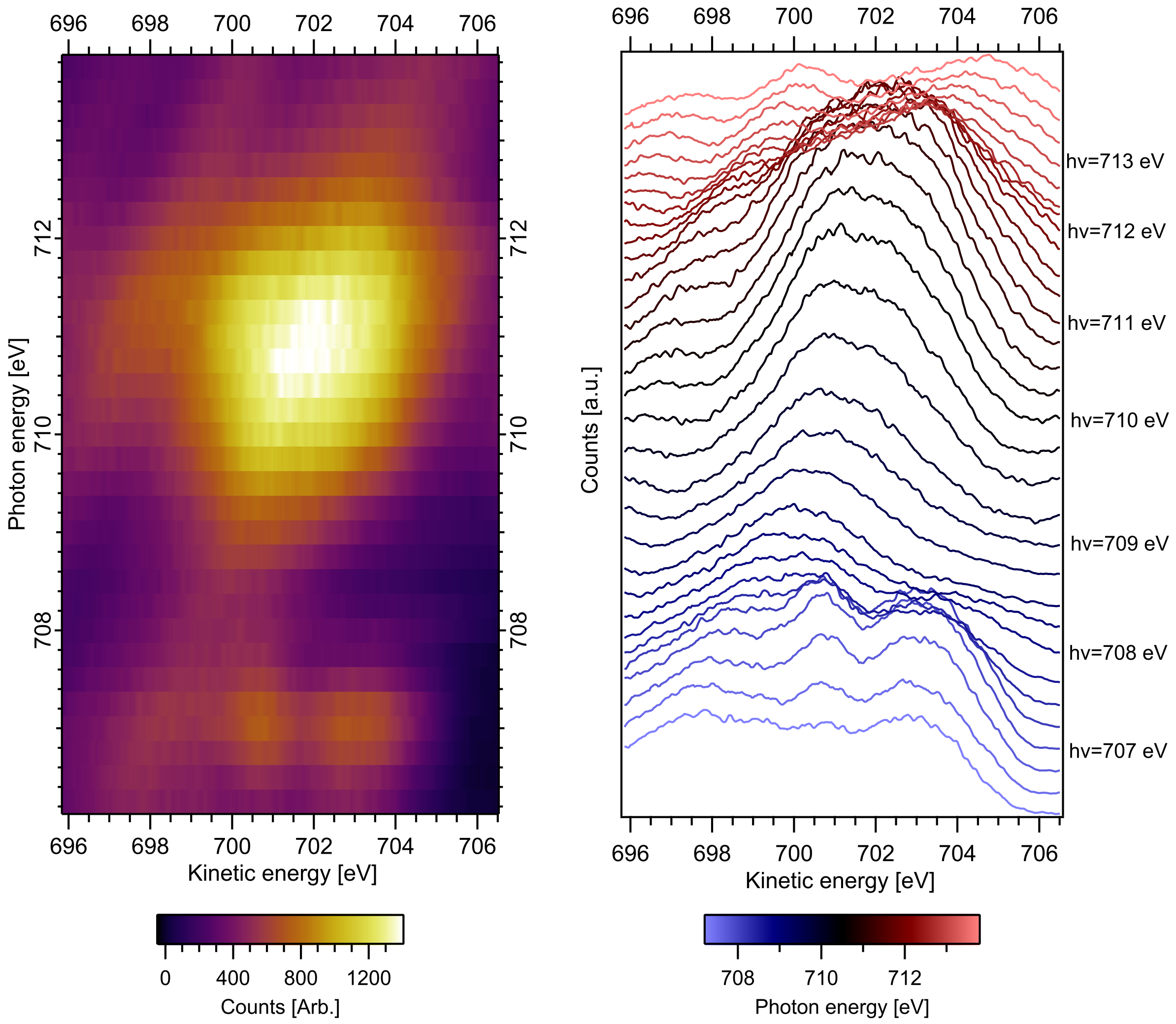

Supplementary Figure 3. $[Fe^{III}(btz)_3]^{3+}$ Fe 2*p* RPES data shown on a kinetic energy axis.

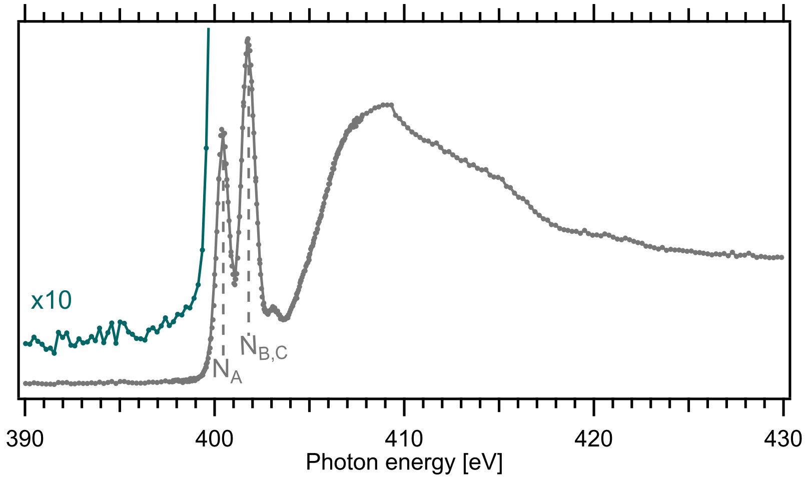

Supplementary Figure 4. $[Fe^{III}(btz)_3]^{3+}$ N 1*s* NEXAFS spectrum.

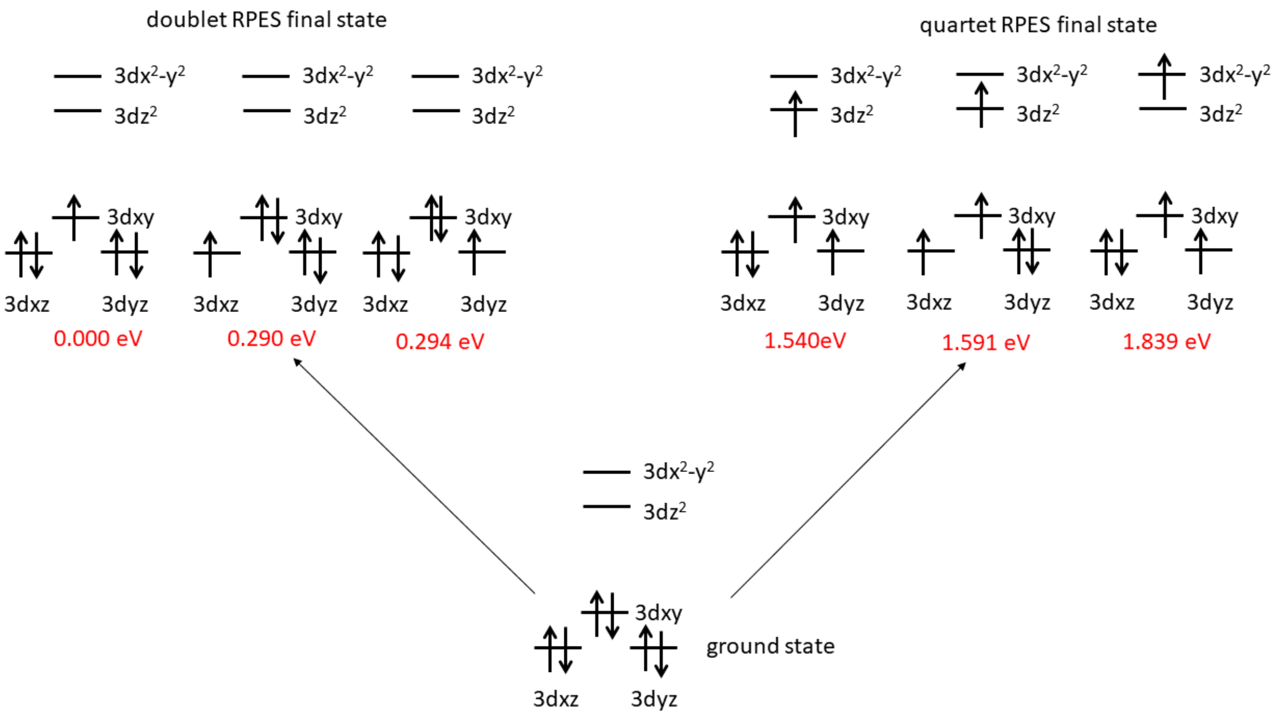

Supplementary Figure 5. Calculated RPES final state electronic configurations for $[Fe^{II}(btz)_2(bpy)]^{2+}$.

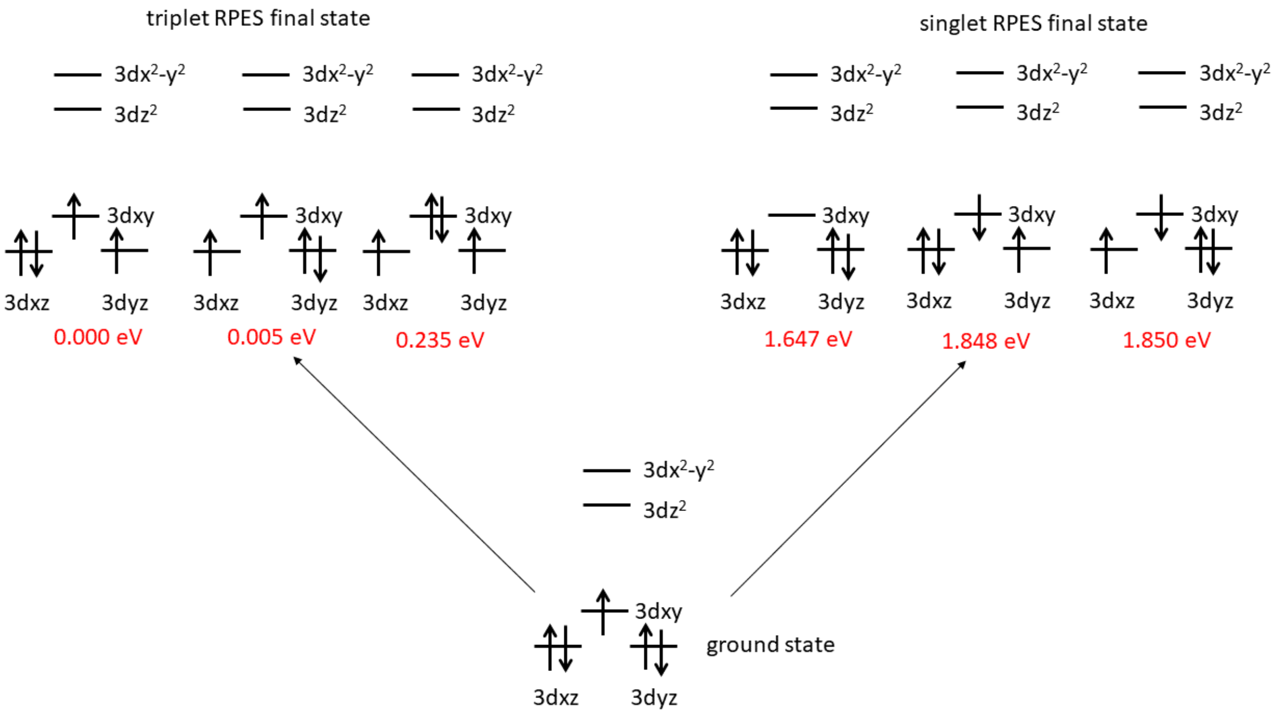

Supplementary Figure 6. Calculated RPES final state electronic configurations for $[Fe^{III}(btz)_3]^{3+}$.

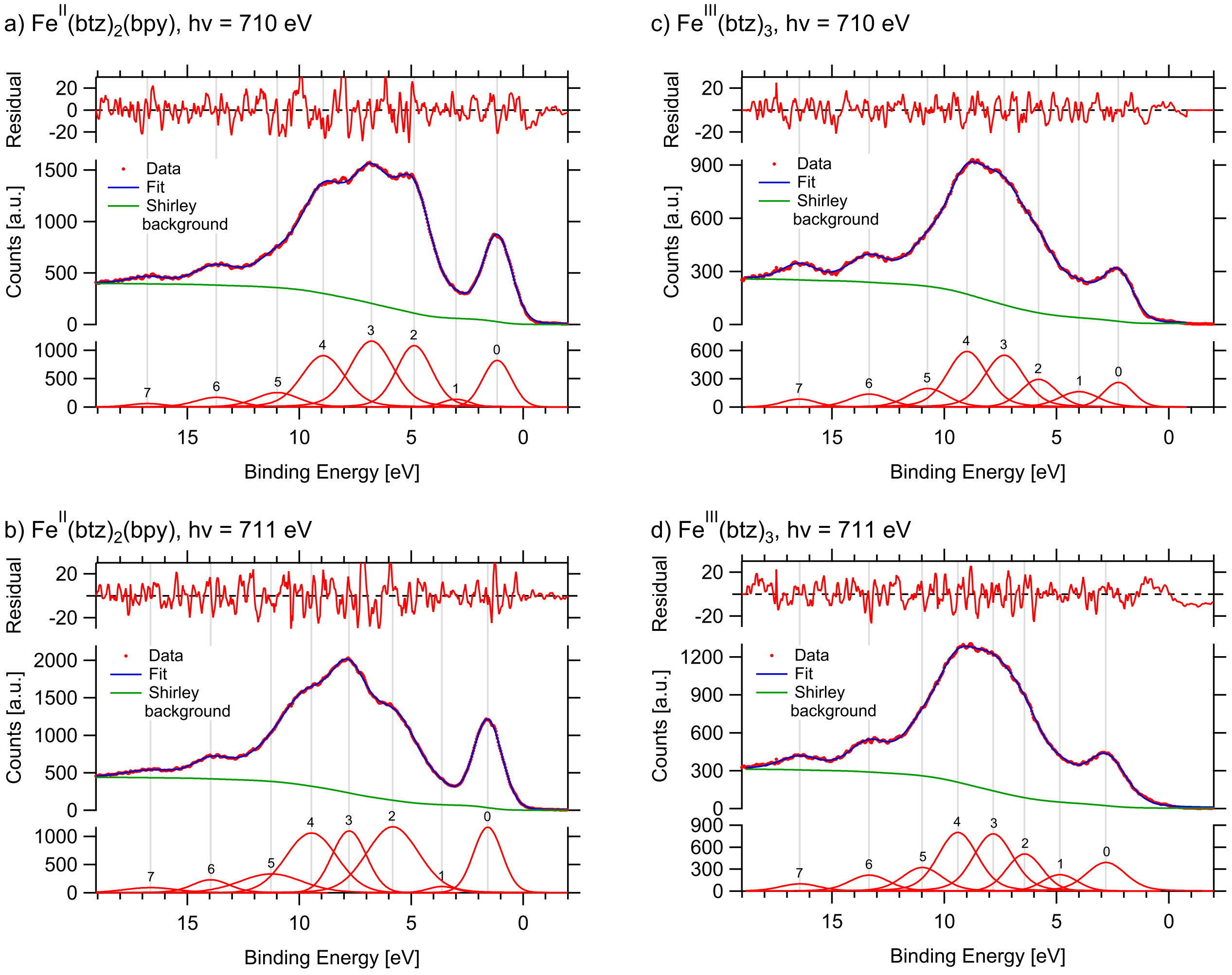

Supplementary Figure 7. Peak fitting of RPES measured at 710 eV and 711 eV photon energy, for both $[Fe^{II}(btz)_2(bpy)]^{2+}$ and $[Fe^{III}(btz)_3]^{3+}$, to extract the binding energy of the t_{2g} peak (peak 0). All other peaks (peaks 1 to 7) are arbitrary to model the background/spectral shape. All peaks are Voigt functions. The positions of peak 0, and associated uncertainties, are listed in Supplemtary Table 2.

Supplementary Table 2. Binding energy (BE) position of the t_{2g} peaks in the RPES, measured at 710 eV and 711 eV photon energy, extracted from curve fitting (peak 0 in Supplementary Figure 7).

Complex	hv [eV]	BE [eV]	BE Uncertainty [eV]
$[Fe^{II}(btz)_2(bpy)]^{2+}$	710.0	1.16	0.03
	711.0	1.58	0.01
$[Fe^{III}(btz)_3]^{3+}$	710.0	2.24	0.14
	711.0	2.8	0.24
